# Supplementary material for: Multistage classification identifies altered cortical phase- and amplitude-coupling in Multiple Sclerosis
Source: Neuroimage. 2022 Dec 1;264:119752. doi: 10.1016/j.neuroimage.2022.119752 (PMC9771829; doi:10.1016/j.neuroimage.2022.119752)
Supplement: Supplementary file 1 [file mmc1.pdf]

Multistage classification identifies altered cortical phase- and amplitude-coupling in Multiple Sclerosis

Marcus Siems, Johannes Tünnerhoff, Ulf Ziemann and Markus Siegel

Supplementary Figures

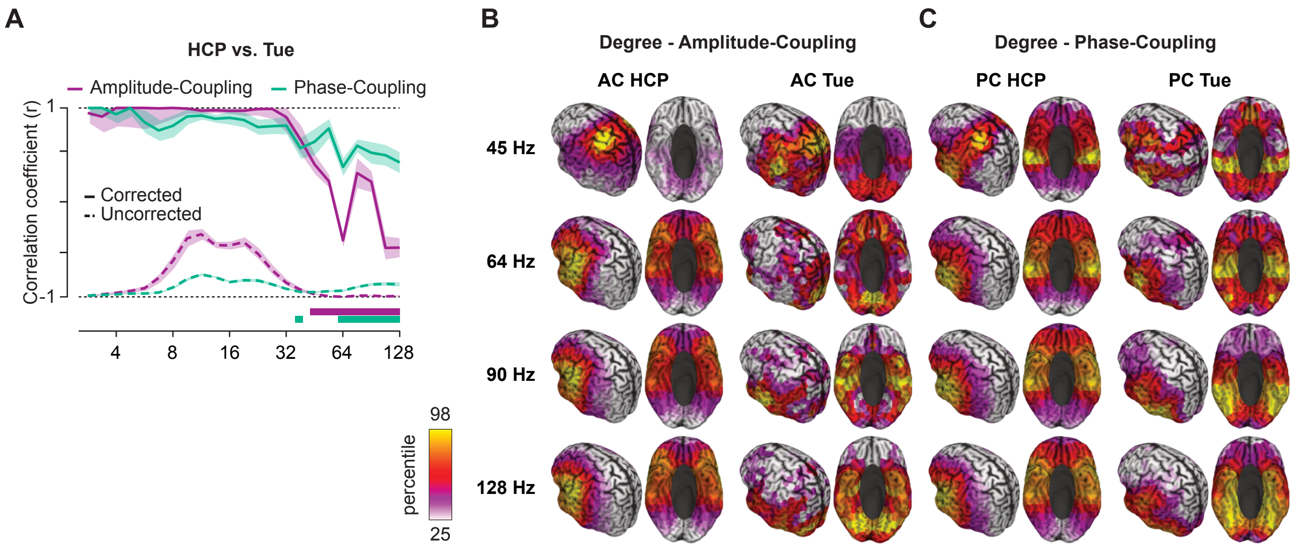

Fig. S1. Comparison of coupling between HCP and Tübingen datasets. (**A**) Raw (dashed lines) and attenuation corrected correlation (solid lines) between connectivity profiles of the HCP (BTI) and the Tübingen (CTF) dataset (healthy controls only). Coupling is compared both for amplitude- (purple line) and phase-coupling (green line). Shaded areas indicate SEM across leave-one-out pseudo-values. Colored bars indicate inter-site correlation significantly smaller than 1 ($r_{corrected} < 1$, $p < 0.05$, FDR-corrected). (**B**) & (**C**) Mean coupling patterns (Degree) for high carrier frequencies (45 – 128 Hz) for (B) amplitude- & (C) phase-coupling for the HCP (left) and Tübingen control subjects (right). The color scale is between the 25th to 98th percentile within each panel.

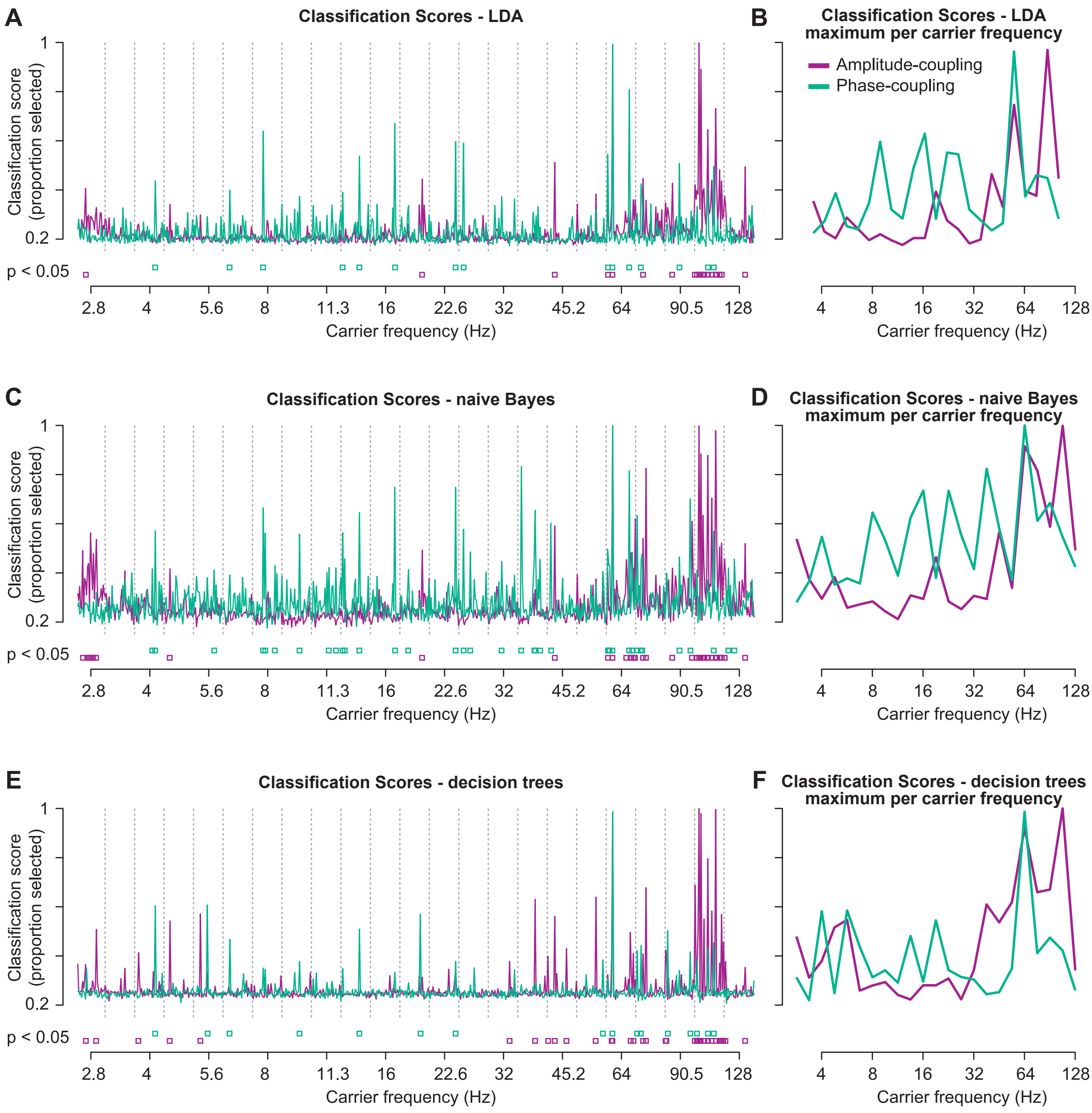

Fig. S2. Comparison between different classification methods. (**A, C, E**) Classification scores for all 30 amplitude- (purple) and phase-coupling (green) components of each carrier frequency for LDA, naïve Bayes, and decision tree classifiers. Purple and green squares indicate significant classification scores for amplitude- and phase-coupling components, respectively ($p < 0.05$, FDR-corrected). (**B, D, F**) Maximum classification scores per carrier frequency for amplitude- (purple) and phase-coupling (green) for LDA, naïve Bayes, and decision tree classifiers. All results are shown as in Fig. 3 for SVM.

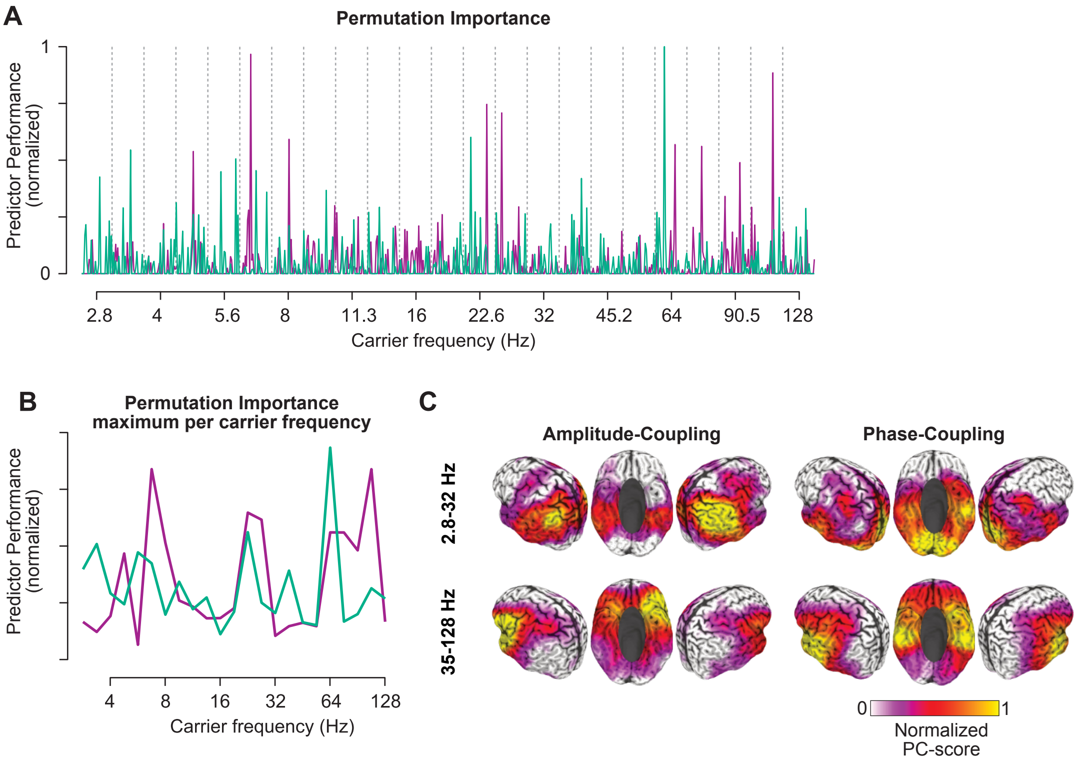

Fig. S3. Classification results from a random forest classifier. (**A**) Weight per component (Permutation Importance) for all 30 amplitude- (purple) and phase-coupling (green) components of each carrier frequency for the classification between patients and control subjects. (**B**) Maximum permutation importance per carrier frequency for amplitude- (purple) and phase-coupling (green). (**C**) Normalized average absolute strength for amplitude- (left) and phase-coupling (right), and for frequencies below (top) and above (bottom) 35 Hz. All components were normalized and scaled according to their respective predictor importance prior to averaging. Colors are scaled between 0 and maximum for each panel. All results are shown as in Fig. 3 & 4 for SVM.
